# Inter-individual variance and cardiac cycle dependency of aortic root dimensions and shape as assessed by ECG-gated multi-slice computed tomography in patients with severe aortic stenosis prior to transcatheter aortic valve implantation: is it crucial for correct sizing?

**DOI:** 10.1007/s10554-012-0123-4

**Published:** 2012-09-18

**Authors:** Lukas Lehmkuhl, Borek Foldyna, Konstantin Von Aspern, Christian Lücke, Matthias Grothoff, Stefan Nitzsche, Jörg Kempfert, Martin Haensig, Ardawan Rastan, Thomas Walther, Friedrich-Wilhelm Mohr, Matthias Gutberlet

**Affiliations:** 1 Department of Diagnostic and Interventional Radiology, University of Leipzig—Heart Centre, Strümpellstrasse 39, 04289 Leipzig, Germany; 2 Clinic of Cardiac Surgery, University of Leipzig—Heart Centre, Leipzig, Germany; 3 Department of Cardiac Surgery, Kerckhoff-Clinic, Bad Nauheim, Germany

**Keywords:** Transcatheter, Percutaneous, Aortic valve, Implantation, Computed tomography, Effective diameter

## Abstract

To evaluate the inter-individual variance and the variability of the aortic root dimensions during the cardiac cycle by computed tomography (CT) in patients with severe aortic stenosis prior to transcatheter aortic valve implantation (TAVI). Fifty-six patients (m/w = 16/40, 81 ± 6.8 years), scheduled for a transapical aortic valve implantation with available preprocedural ECG-gated CT were retrospectively included. The evaluation included sizing of the aortic annulus and the aortic sinus, measurements of the coronary topography, aortic valve planimetry and scoring of calcification. The new defined aortic annulus sphericity ratio revealed a mostly elliptical shape with increasing diastolic deformation. The calculated effective diameter (ED), determined from the annulus’ lumen area, turned out to be the parameter least affected from cardiac cycle changes while systolic and diastolic annulus dimensions and shape (diameter and area) differed significantly (*p* < 0.001). In about 70 % of the patients with relevant paravalvular leaks the finally implanted prosthesis was too small according to the CT based calculated ED. The ostial height of the coronaries showed a high variability with a critical minimum range <5 mm. The degree of the aortic calcification did not have an influence on the aortic annulus deformation during the cardiac cycle, but on the occurrence of paravalvular leaks. The aortic root anatomy demonstrated a high inter-individual variability and cardiac cycle dependency. These results must be strongly considered during the patient evaluation prior to TAVI to avoid complications. The systolic effective diameter, as measured by ECG-gated CT, represents an appropriate parameter for sizing the aortic annulus.

## Introduction

Catheter-based antegrade (transapical) and retrograde (transfemoral) aortic valve implantation are promising treatment methods for patients with severe aortic stenosis (AS) and high perioperative risk. These transcatheter approaches have shown promising postoperative results because they have a significantly lower perioperative risk [1, 2] and are already considered to be routine procedures in experienced facilities.

Nevertheless, these approaches have the disadvantage of not allowing direct visualization of the aortic valve and the aortic root during the interventional procedure. For this reason, pre- and intra-operative imaging is crucial for procedural success. Pre-operative imaging modalities that are suitable and widely used include transesophageal echocardiography (TEE), multislice computed tomography (CT) and, less commonly, magnetic resonance imaging [3, 4]. Additionally, intra-operative imaging modalities such as fluoroscopy, TEE and 3D-rotational angiography can be used [5].

While open heart surgery allows direct inspection and sizing of the aortic root and annulus, minimally invasive procedures require that anatomical details are known prior to the procedure to allow adequate preoperative planning, prosthesis choice and patient selection. In TAVI procedures, the aortic annulus size and the distances of the coronary ostia to the aortic annulus, the ostial height, are important preoperative parameters.

In recent years, cardiac CT has been reinforced as a promising non-invasive imaging modality for the assessment of the aortic root [6, 7]; however, little is known about the inter-individual aortic root anatomy and the influence of the cardiac cycle on the dimensions of the aortic root. A study published by de Heer et al. [8] described aortic root changes during the cardiac cycle in patients without aortic root disease. However, the study by Bertaso et al. is the only one that describes the dynamic changes in the aortic annular dimensions in patients with AS. The issue is that this analysis was based on the assumption that the aortic annulus maintains its ellipsoid shape during the entire cardiac cycle and simply included a minimum and maximum diameter [9]. Therefore, the aim of our study was to use a comprehensive CT analysis to evaluate the inter-individual aortic root anatomy and its variability during the cardiac cycle in patients with severe AS prior to TAVI, including different parameters, which may be less affected by changes of aortic root shape during the cardiac cycle.

## Materials and methods

### Study population

Patients who were scheduled for TAVI and an available preprocedural ECG-gated cardiac CT were retrospectively included in this study. TAVI was considered for patients with severe, symptomatic AS, a calculated risk of mortality ≥15 % (according to the logistic EuroScore), and a risk of mortality ≥10 % (according to the Society of Thoracic Surgeons’ score). All risk calculations were performed individually while considering other comorbidities. Patients with a life expectancy of less than 1 year were not considered for TAVI. The exclusion criteria were incomplete CT data, inadequate arterial contrast enhancement below 200 Hounsfield units in the ascending aorta, a heart rate exceeding 110 beats per minute and massive artifacts due to implants. No beta-blockers were administered due to severe AS in all patients. Furthermore, intraoperative and postprocedural TEE data were included in the analysis.

### CT protocol and image analysis

All scans were performed on a 64-row CT (Brilliance 64, Philips Medical Systems, Cleveland, Ohio, USA), which captured the entire heart using retrospective ECG gating. Patients were examined in the supine position during a single breath hold. Intravenous administration of 70 ml of nonionic iodinated contrast medium (Iopromide, 370 mg iodine per ml, Ultravist 370, Schering, Berlin, Germany) was provided at a flow rate of 4 ml/s followed by 60 ml saline flush. The CT scan began by bolus tracking in the left atrium and was performed in the caudocranial direction. A collimation of 64 × 0.625 mm at a rotation time of 0.4 s (Pitch 0.2) was used. Tube current and voltage were 800 mAs and 120 kV, respectively. The images were reconstructed at a slice thickness of 0.67 mm and an increment of 0.4 mm using a soft tissue reconstruction algorithm (Table 1). The retrospectively gated image data were reconstructed into 10 cardiac phases that each represented 10 % of the R–R interval starting at the beginning of the R–R interval (Fig. 2).

**Table 1:** Scan protocol parameters

Parameter	Value
Peak voltage (kVp)	120
Rotation time (s)	0.4
Effective tube load (mAs)	800
Collimation (mm)	64 × 0.625
Slice thickness (mm)	0.67
Increment	0.4
Table feed (mm)	8
Pitch factor	0.2
FOV (cm)	28
CTDIvol (average) (mGy)	46.8 ± 3.6
DLP (average) (mGy × cm)	888.6 ± 46.3
Effective dose (average) (mSv)*	12.4
Contrast agent	Iopromide (370 mg iodine/ml)
Injection flow (ml/s)	4.0

*SD* standard deviation, *kVp* peak kilovolt, *s* seconds, *mAs* milliampere × seconds, *mGy* milligray, *mSv* millisievert

* Estimated from DLP with conversion factor k = 0.014 mSv/mGy × cm

All of the image post-processing and analysis were performed on a commercially available medical workstation (Philips Extended Brilliance Workspace V 3.5.0.2254, Comprehensive Cardiac and CT Viewer, Philips Medical Systems, Best, Netherlands).

### Definition of heart phases

All of the measurements were performed separately in systole and diastole. Usually, it is common in retrospectively gated CT to define the systolic and diastolic phases with fixed percentages of the R–R interval (e.g., systole 30 %, diastole 70 %). However, as the phases of the cardiac cycle depend on the heart rate, we decided to use the physiological definition of systole and diastole as defined visually by using the time-based cine mode. Diastole was defined as the cardiac phase when the mitral valve was completely opened, the aortic valve was closed and the left ventricle was maximally filled. Systole was defined as the time interval when the mitral valve was closed, the aortic valve was completely open, and the left ventricular volume was minimal. The resultant percentage of the R–R-interval was noted for the visually defined systolic and diastolic phases.

### Definition of anatomical landmarks and effective diameter

**Fig. 1:**
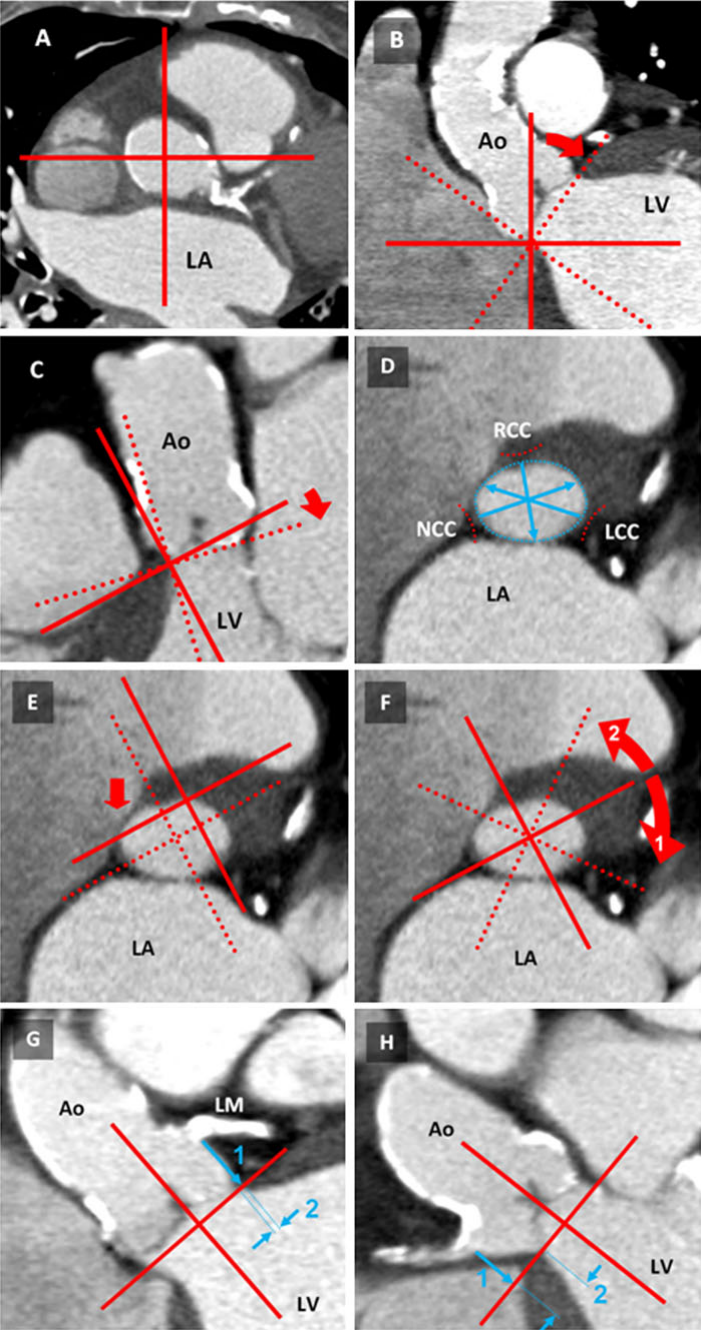
Aortic root measurement procedure. **A** To begin, the crosshair was placed in the aorta in a transverse section (here at the level of the sino-tubular junction) **B** In the coronal view, the crosshair was moved to the most basal attachment of any cusp (here: NCC). The crosshair was rotated until one plane reached the corresponding part of the opposite cusp (here: LCC). **C** In the sagittal view, the same plane was adapted to the next basal cusp attachment (here: RCC). Control of the plane position obtained by scrolling through the image stack. **D** In the oblique transverse view, the aortic annulus was displayed and used for diameter measurements including the effective diameter. **E** For coronary ostia measurements, the crosshair was placed in the center of the aortic annulus and rotated until (**F**) the coronary ostia of the LCA (1) and RCA (2) appeared in the corresponding coronal or sagittal view. **G** In the plane displaying the LCA ostium, the distance to the aortic annulus was measured (1). Additionally, LCA to LSC (2) was assessed. **H** The RCA ostium distance (1) and the RCA LSC (2) were measured similarly as in step **F**. *Ao* ascending aorta, *LV* left ventricle, *LA* left atrium, *LM* left main, *RCA* right coronary artery, *RCC/LCC/NCC* right-/left-/non-coronary valve cusp, *LSC* lateral shift of the coronary ostia

The aortic root was defined as the part of the aorta from the aortic annulus to the sinotubular junction, which included the aortic annulus, cusps, sinus, sinotubular junction and the coronary ostia. The aortic annulus was defined as a virtual plane at the level of the basal attachments of the aortic cusps [10]. The sinotubular junction was defined as the section with the lowest lumen area between the aortic sinus and the ascending aorta. The effective diameter (ED) was defined as the diameter of a virtual circle with the same cross-sectional area as the vessel in a particular section of interest (Fig. 1D) [5, 11]:

$$ {\text{ED}} = 2 \times \sqrt {\left( {{\text{cross-sectional}}\,{\text{area}}/\pi } \right)} . $$

### Aortic sinus analysis

To estimate the relationship of the coronary ostia to the aortic annulus, the distances between the aortic annulus and the proximal portion of the right and left coronary ostia were measured perpendicular to the aortic annulus plane (Fig. 1G–H). The lateral shift of the coronary ostia to the inner border of the aortic annulus (LSC, Lateral Shift of the Coronary artery ostium) was measured in a plane parallel to the aortic annulus (Fig. 1G–H). Furthermore, the ED of the sinotubular junction was estimated using a vessel path-based curved multiplanar reconstruction.

### Aortic valve analysis

The analysis of the aortic valve included the aortic annulus, the aortic valve area (AVA), the regurgitant orifice area (ROA) and the amount of aortic valve calcification.

#### Aortic annulus diameter, cross-sectional area and calculation of the effective diameter (ED)

The assessment of the aortic annulus contained measurements of three separate distances at the level of the aortic annulus between the basal attachment of the aortic cusps and the opposite intercommissural region of the aortic root wall (Fig. 1D). The opposite intercommissural region was defined as the midpoint of the partial circumference between the basal attachments of the remaining two aortic cusps. The exact position was controlled by scrolling through adjacent parallel planes. Additionally, the lumen area of the aortic annulus was measured at the same level to calculate the ED.

#### Aortic annulus sphericity

To describe the aortic annulus sphericity and its varying shape during the cardiac cycle, we introduced the anatomical aligned aortic annulus sphericity ratio (AASR). The AASR was defined as the ratio of the largest distance of the 3 measurements between the basal attachment of the aortic valve cusps and the opposite intercommissural region (D_L_) divided by the smallest distance (D_S_):

$$ AASR = \frac{{ D_{L} }}{{ D_{S} }}. $$

An AASR of 1.0 signifies an aortic annulus with an ideal circular shape. An AASR higher than 1.0 indicates an aortic annulus with increasing deformation (Fig. 4A). We did not use the eccentricity ratio described by Doddamani et al. [12] for the left ventricular outflow tract because we believe that it does not adequately represent the tripartite anatomy of the aortic valvular complex.

### Prosthesis oversizing and rate of paravalvular leaks

According to the clinical standard in our institution, a 2 mm prosthesis oversizing was consistently aimed in relation to the annulus size measured by intraoperative TEE [13]. Patients with a postprocedural paravalvular leak, classified as moderate or severe and confirmed through TEE, were compared to those without a paravalvular leak (none or minimal). Therefore, the intraoperative TEE based-measured annulus diameter and the preoperative CT-based systolic ED were substracted from the finally implanted prosthesis size.

#### AVA, ROA and aortic valve calcification

AVA and ROA were both measured planimetrically at the level of the minimal systolic outflow area and at the level of the maximal diastolic orifice area in the diastolic phase, respectively.

Aortic valve calcification was quantified by calcium scoring, which was analogous to the Agatston score for coronary arteries (ASE, Agatston Score Equivalent). Calcification was measured to estimate its influence on the change of the aortic annulus shape during the cardiac cycle. Calcium quantification was performed as a total score in the area between the aortic annulus and the sino-tubular junction, excluding the calcification of the coronary arteries. Aortic valve calcium scores were measured by multiplying the lesion area by an attenuation factor derived from the maximal Hounsfield units within the area, as previously described by Agatston et al. [14], using a detection threshold of 130 HU.

### Statistical analysis

Quantitative variables are expressed as the mean ± standard deviation. Significance was defined as *p* < 0.05 and calculated using a paired *t* test. Linear regression analysis was performed using Pearson’s correlation coefficient. The null hypothesis was tested using a t-distribution. All statistical analyses were performed using commercially available software (SPSS 17 for Windows, SPSS Inc., Chicago, IL, USA).

## Results

### Implanted valves and study population

ECG-gated CT data prior to a scheduled percutaneous valve implantation were available for evaluation in 56 patients (m/f = 16/40, mean age 81.6 ± 6.8 years). The mean heart rate during image acquisition was 77.8 ± 12.8/min (range 45–108) (Table 2).

**Table 2:** Clinical patient data (n = 56) and implanted prostheses

Parameter	Value
Male/female	16/40 (29/71 %)
Age, years ± SD (range)	Mean 81.6 ± 6.8 (60–94), m: 76.9 ± 6.9, f: 83.5 ± 5.9
AVA, mm^2^ ± SD (range)	90.7 ± 14.2 (57–118.1)
ROA, mm^2^ ± SD (range)	3.5 ± 6.3 (0–32)
Ejection fraction, % ± SD	50 ± 18
TAVI (Edwards Sapien)	Total n = 43 (Ø 23 mm: n = 11; Ø 26 mm: n = 32)
TAVI (Ventor Embracer)	Total n = 2 (Ø 23 mm)
TAVI (Corevalve)	Total n = 2 (Ø 29 mm: n = 2)
Relevant paravalvular leakage	10 (Edwards Sapien: n = 9/43, Corevalve: n = 1/2, Ventor: n = 0/2)
Conventional surgery	n = 6
No valve implantation	n = 3

*SD* standard deviation, *AVA* aortic valve area, *ROA* regurgitant orifice area, *TAVI* transcatheter aortic valve implantation

Valve replacement was performed in 53/56 (95 %) patients. Three procedures were canceled (5 %) because of adverse clinical conditions. Six (6/53 = 11 %) patients did not fulfill the inclusion criteria for TAVI based on the pre-interventional screening process; therefore, these patients received a conventional aortic valve replacement. The patients that did not fulfill the inclusion criteria presented either an inappropriate annulus size (n = 1) or a EuroScore that was too low (n = 5). Therefore, 47 out of the 53 treated patients received a percutaneous valve implantation. The majority of these patients (n = 43) received a transapically inserted Edwards-Sapien prosthesis (Carpentier-Edwards Lifesciences, Irvine, CA, USA) using the two commercially available valve sizes. The larger valve size (26 mm) was implanted in 32 patients, and the smaller-sized valve (23 mm) was implanted in 11 patients. A Ventor-Embracer (23 mm) prosthesis (Ventor Technologies, Netanya, Israel) was used in two patients. Two other patients (4 %) had an annulus size larger than 26 mm and therefore received a 29 mm CoreValve prosthesis (CoreValve Inc, Irvine, CA, USA) delivered transfemorally.

Intraoperative TEE-Sizing measurements of the annulus and postprocedural TEE were available for further analysis in 47/56 and 45/56 patients, respectively. In two patients, no valid information about the presence of a postprocedural leak was available. Relevant (moderate and severe) paravalvular leaks after valve implantation were present in 10/45 patients (Edwards Sapien: 9/43, Corevalve: 1/2). One patient with severe aortic leakage underwent a valve-in-valve procedure. Due to the small number of post-procedural leaks, the influence of the valve type on the leaks could not be assessed.

### Heart phase definition by the R–R interval versus a physiological definition

**Fig. 2:**
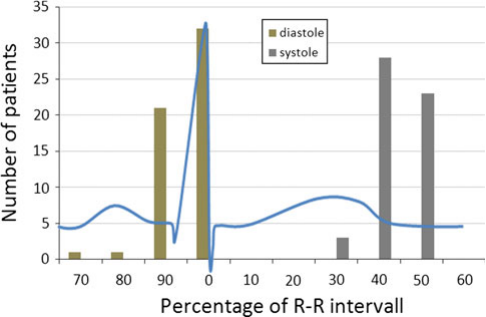
Heart phase definition based on a physiologic definition versus a fixed R–R interval. In most of the patients the reconstruction interval for an end-systolic or end-diastolic reconstruction had to be changed when a physiologic definition was used instead of a fixed R–R interval. Systole was usually found at 40–50 % of the ECG R–R interval and the diastole at 90–100 %

In most of the patients, the reconstruction interval for an end-systolic or end-diastolic reconstruction had to be changed when a physiologic definition was used instead of a fixed R–R interval. In the majority of patients, the end-systolic phase was at 40–50 % of the ECG R–R interval, and the end-diastolic phase was identified at 90–0 % of the R–R interval (Fig. 2).

### Aortic sinus analysis

#### Effective diameter (ED) of the sinotubular junction

The mean ED of the sinotubular junction did not differ significantly between systole and diastole, with a mean difference of 0.5 mm ± 0.8 (*p* = 0.22) (Table 3).

**Table 3:** Aortic root measurements

Aortic root distances	Mean values (mm ± SD), (range)		Mean Diff.	CI (95 %)
	Systole	Diastole		
ED sinotubular junction	27.4 ± 3.2 (22.2–38.4)	27.2 ± 3.2 (22.5–38.7)	0.6 ± 0.8	−1.0 to 0.4
Annulus to LCA	12.2 ± 2.5 (5.3–18.8)	12.9 ± 2.5 (5.1–19.7)	1.3 ± 1.0	−0.8 to 0.1
Annulus to RCA	12.2 ± 3.0 (3.7–19.6)	12.9 ± 2.9 (6.6–20.7)	1.4 ± 1.1*	−1.1 to 0.2
lateral shift (LSC) of LCA	2.4 ± 1.8 (0–6.9)	2.6 ± 1.9 (−1.8 to 7)	1.0 ± 0.9	−5.3 to 0.2
lateral shift (LSC) of RCA	3.9 ± 2.2 (0–11.5)	4.2 ± 2.2 (0–13.1)	1.5 ± 1.2	−0.8 to 0.2

*SD* standard deviation, *CI* confidence interval, *ED* effective diameter, *LCA/RCA* left/right coronary artery, *LSC* Lateral Shift of the Coronary artery ostium

* *p* < 0.01

#### Distance of the aortic annulus to the coronary artery ostia (Ostial Height)

The distances from the aortic annulus to the coronary ostia were, on average, more than 12 mm. However, the data revealed that small distances of the coronary ostia, indicating a potential risk for coronary obstruction during the implantation procedure, can be found for both, the LCA and RCA ostia (minimum distance LCA = 5.1, RCA = 3.7). In the pairwise analysis, the mean distance between the RCA ostium and the aortic annulus differed significantly in the systolic and diastolic phases, with a mean difference of 1.4 ± 1.1 mm (*p* = 0.007). No significant difference was observed between the systolic and diastolic phases for an LCA distance to the aortic annulus, with a mean difference of 1.3 ± 0.96 mm (*p* = 0.112).

#### Lateral shift (LSC) of the coronary ostia to the inner border of the aortic annulus

The aortic sinus and the position of the coronary ostia relative to the aortic annulus showed a high inter-individual variability. The mean distances from the LSC to the inner border of the aortic annulus for the LCA and the RCA in systole were 2.4 mm (2.6 mm diastole) and 3.9 mm (4.2 mm diastole), respectively. The LSC values revealed patients that presented a rather tubular sinus shape without any lateral shift of the coronary ostia. The LSC of the right and left coronary ostia did not differ significantly between the systolic and diastolic phases (right: *p* = 0.280; left *p* = 0.339) with mean differences of 1.5 ± 1.2 mm and 1.0 ± 0.9 mm for the right and left coronary ostium, respectively.

### Aortic valve analysis

#### Diameters of the aortic annulus

**Fig. 3:**
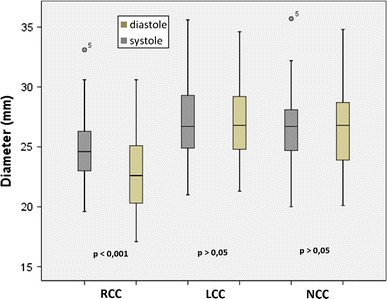
Aortic annulus diameter. The mean distances between the basal attachment of the aortic valve cusps and each contralateral intercommissural region for the right-, left- and non-coronary cusp. Significant differences were found comparing the right coronary cusp (RCC) in the systolic and diastolic phases (*p* < 0.001) and the RCC to the left (LCC) and non-coronary cusp (NCC) (*p* < 0.001)

The mean distance between the basal attachment of the RCC and the opposite intercommissural region was significantly shorter than the corresponding LCC and NCC distances (*p* < 0.001). Moreover, only the RCC distance differed significantly between systole and diastole (*p* < 0.001) (Table 4; Fig. 3).

**Table 4:** Aortic valve measurements

Aortic valve parameters	Mean values(mm ± SD), (range)		Mean Diff.	CI (95 %)
	Systole	Diastole		
Annulus diameter				
RCC	24.8 ± 2.9 (19.6–33.1)	23.0 ± 3.2 (17.1–30.6)	2.2 ± 1.6**	1.3–2.4
LCC	27.1 ± 3 (21–35.6)	27.0 ± 3.0 (21.3–34.6)	1.6 ± 1.2	−0.5 to 0.7
NCC	26.4 ± 3.3 (16.2–35.7)	26.5 ± 3.4 (20.1–34.8)	2.0 ± 1.8	−0.6 to 0.8
Mean (RCC, LCC, NCC)	26.2 ± 3.1 (19.6–35.7)	25.5 ± 3.7 (17.1–34.8)	0.7 ± 2.3**	0.3–1.0
ED	25.8 ± 2.6 (20.5–32.7)	25.2 ± 3.0 (18.7–32.3)	0.6 ± 1.2**	0.2–0.9
Intraprocedural TEE	22.6 ± 1.5 (20.0–25.00)			
AASR**	1.12 ± 0.1 (1.0–1.2)	1.20 ± 0.1 (1.0–1.4)	−0.08 ± 0.09**	−0.11 to 0.06

*SD* standard deviation, *CI* confidence interval, *ED* effective diameter, *RCC/LCC/NCC* right-/left-/non-coronary cusp, *LCA/RCA* left/right coronary artery, *AASR* aortic annulus sphericity ratio

** *p* < 0.001

The ED of the aortic annulus differed significantly (*p* = 0.001) between the systolic and diastolic phases with a mean difference of 0.6 (±1.2) mm.

The correlation between the ED and the distances between the basal attachment of the aortic valve cusps to each opposite intercommissural region in the systolic phase was very high for the RCC and LCC (r = 0.90 and r = 0.92, *p* < 0.01) and lower for the NCC (r = 0.78, *p* < 0.01). In the diastolic phase, the distances between the basal attachment and each opposite intercommissural region correlated very strongly for all three cusp diameters (r = 0.92 each, *p* < 0.01).

#### Aortic annulus sphericity ratio (AASR)

**Fig. 4:**
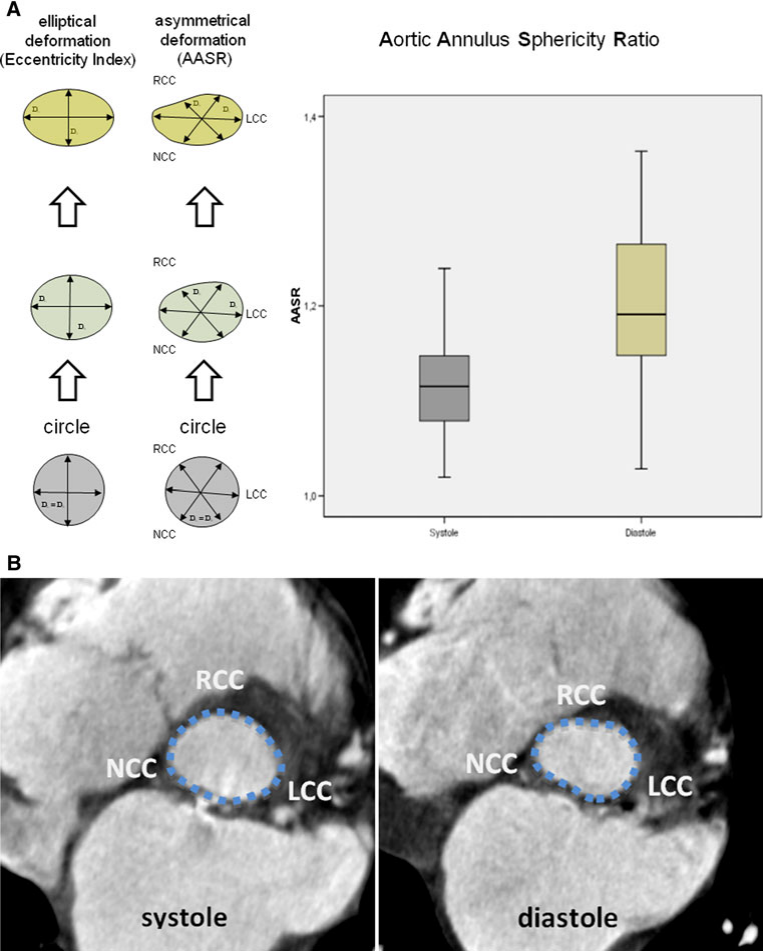
**A** Deformation of the aortic annulus shape in systole and diastole: Note the difference of the Eccentricity Index and the newly introduced AASR (Aortic Annulus Sphericity Ratio). The degree of asymmetry of the aortic annulus plane during systole and diastole was found to increase in diastole (*p* < 0.001). An AASR and Eccentricity Index of 1.0 stands for a shape of the aortic annulus, which corresponds to an ideal circle. An AASR higher than 1.0 indicates increasing asymmetrical deformation. **B** Example of an asymmetrical deformation of the aortic annulus: The systolic shape of the aortic annulus is approximately elliptical while the diastolic deformation especially affects the RCC portion of the annulus

The AASR, which is a surrogate for aortic annulus asymmetry, was significantly different within the cardiac cycle (*p* < 0.001). In the systolic phase, the mean AASR was 1.12 (±0.05, range 1.02–1.24). Increasing deformation of the aortic annulus was observed due to an increasing AASR in the diastolic phase with an average of 1.20 (±0.08, range 1.03–1.36) (Table 4; Fig. 4A/B). There was no significant correlation between the AASR and the rate of paravalvular leaks (r = 0.2, *p* = 0.15).

#### Prosthesis oversizing and rate of paravalvular leaks

**Fig. 5:**
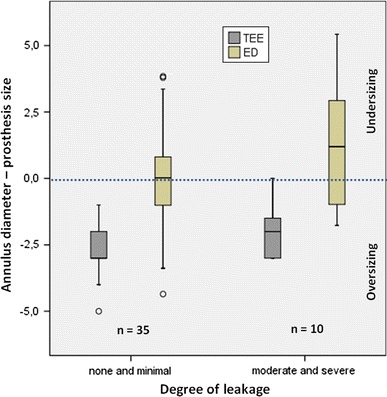
Prosthesis oversizing and rate of paravalvular leaks. This *graph* shows the degree of prosthesis oversizing derived from TEE (*grey boxplot*) and CT-based effective diameter (ED, *green boxplot*), grouped by the degree of paravalvular leakage after TAVI. Values below the *blue neutral line* indicate a prosthesis oversizing (in mm), while values above this *line* indicate a prosthesis size smaller than the aortic annulus

Based on our measurements of systolic ED in CT, the goal of having 2 mm oversizing was not achieved in the majority of the patients (40/47). The CT-based ED measurements revealed that no 2 mm prosthesis oversizing was accomplished in any patient (10/10) with a relevant paravalvular leak (Fig. 5). In patients without a relevant leak, this discrepancy was significantly lower (*p* < 0.01). No relevant paravalvular leaks were present in patients with a CT-based oversizing of 2 mm or more.

#### Degree of aortic valve calcification

The mean Agatston Score equivalent (ASE) of the aortic valve was 3,294.5 (±2,014.2, range 0–7,518.2). In the majority of patients, the degree of valvular calcification was estimated as “moderately calcified” (no calcification 3.7 %, mild calcification 29.6 %, moderate calcification 42.6 %, heavy calcification 24.1 %; median classification: moderate calcification). Only three patients did not present any calcification of the aortic valve. Patients with a relevant paravalvular leak (moderate and severe) had a significantly higher degree of calcification than those without a relevant (none and minimal) leak (*p* = 0.047).

To determine whether aortic root calcification influenced the sphericity of the aortic annulus during the heart cycle, the AASR difference in the systolic and diastolic phases was correlated to the ASE but was not relevant (r = −0.05, *p* = 0.72, 1. ASE quartile: mean AASR difference = 0.12, 4. ASE quartile: mean AASR difference = 0.10).

## Discussion

The number of TAVI procedures has increased significantly in the last several years [1, 2]. The high procedural success rate of TAVI is a consequence of optimized patient selection by pre-operative imaging. However, several complications have been reported. Paravalvular leaks are frequent and a strong, significant predictor of in-hospital mortality. In addition, aortic annulus sizing based on TEE, in comparison with CT, is associated with a higher leakage rate [15]. One important reason for the resulting leakage is the inaccurate aortic annulus sizing and consequential prosthesis mismatch. Recently published studies have focused on the comparison of CT and TEE measurements [6, 7, 16, 17]; however, little attention has been given on the inter-individual differences in anatomy of the aortic root or the deformation of the aortic root during the cardiac cycle and the concomitant change of aortic root dimensions.

In this regard, our study can be summarized as follows: In the majority of patients, the geometrical shape of the aortic annulus could be best described as an ellipsoid with increasing asymmetrical deformation during diastole. The systolic and diastolic effective diameter was not affected by the shape of the annulus, and the systolic phase provides the largest annulus diameter. The degree of aortic valve calcification does not have an influence on aortic annulus deformation during the cardiac cycle, but on the occurrence of paravalvular leakages, as previously described [18]. Small distances to the coronary ostia, which indicate a potential risk of coronary obstruction during the implantation procedure, could be found for both the LCA and the RCA ostium. The aortic sinus showed a high anatomical variability with some patients having a notably tubular shaped sinus without any lateral shift of the coronary ostia.

The geometrical shape of the aortic annulus has been previously described as an ellipsoid [6, 9, 12, 19] and was confirmed by the AASR in our study. In our patients, the mean diameter between the RCC and the opposite intercommissural region was significantly lower than the LCC and NCC diameters. Additionally, we found an asymmetrical, cardiac cycle dependent deformation of the aortic annulus, which was almost exclusively identified by a diameter change between the RCC and the opposite intercommissural region, but there was not a significant change in the diameter between the NCC, respectively LCC to the contralateral side. These findings are partially in agreement with the results of a previous study by Bertaso et al. [9], which described the dynamic change of the aortic annulus during the cardiac cycle in a comparable patient population. However, doubt remains regarding whether Bertaso et al. detected the entire extent of the change because only the minimum and maximum diameters were included. As expected, this asymmetrical diastolic deformation also affected the lumen area, as represented by the ED of the annulus, which resulted in significantly different measurements for the systolic and diastolic phases.

We thus conclude that the usually performed TEE measurements, based on simple 2D image data sets and the assumption of a circular annulus, do mostly not accurately capture the maximum diameter. Additionally, we conclude, that the asymmetrical shape of the aortic annulus cannot be reliably assessed by a single diameter, neither by CT nor by TEE. Even more, TEE measurements usually performed in the midoesophageal long axis-view seem to capture the aortic annulus closely to the region of the smallest diameter. The differences in the CT based annulus diameter between systole and diastole were found to be up to 2.2 ± 1.6 mm for the RCC diameter and 2.0 ± 1.8 mm for the NCC diameter. Additionally, differences of up to 4 mm were found between the smallest and the largest mean diameter during the cardiac cycle. This demonstrates that single 2D measurements either performed by TEE or CT could result in an error exceeding 2 mm. So far there are only a few prosthesis sizes available and a 2 mm error has a strong impact on correct prosthesis selection.

This deviation seems to have a strong clinical impact in view to the high paravalvular leakage rate of 18 % in our study population. With regard to paravalvular leaks, a 2 mm oversizing was intended to be consistently applied according to the intraoperative TEE measurements. Nevertheless, according to our findings using the CT based ED, no oversizing was actually achieved with the 2D TEE approach in 70 % of patients with a relevant paravalvular leak. This might indicate that a larger than the selected prosthesis would have been potentially a better solution. However, it is speculative whether a selection of the prosthesis based on the systolic CT-based ED would have reduced the number of paravalvular leaks. Moreover, the limited number of available valve sizes at the beginning of our study did not allow for an individual adaption to patient’s anatomy. But this will change as soon as more sizes will be on the market.

It is assumed that 2D-TEE is methodically inferior to ECG-gated CT, which is a 3D imaging method, in the assessment of the aortic annulus diameter [7]. In addition, TEE based annulus sizing has recently been identified as a predictor for post-operative paravalvular leakage [15].

A correct definition of the annulus plane and the cardiac phase is mandatory to perform reliable annulus sizing. In the recent literature, studies that compared CT and TEE/TTE measurements of the aortic annulus diameter describe both the negative [7, 16] and positive mean differences [6, 17] between both methods. These studies may be limited by an insufficient capture of the individual anatomy because the measurements did not seem to be sufficiently aligned with the three-dimensional anatomy of the aortic annulus. Additionally, the measurements were performed in cardiac phases that were defined according to fixed percentages of the R–R interval and did not take into account the actual functional state of the valve and ventricle [6, 7, 16]. One of the cited studies was conducted without any ECG-triggering of the CT image acquisition [17]. Our results showed that the reconstruction interval for end-systolic or end-diastolic images had to be individually changed for each patient when a physiologic definition was used. Moreover, we found that the cardiac cycle significantly influenced the aortic root dimensions.

The ED turned out to be a suitable parameter for the assessment of the aortic annulus because it was unaffected by the annulus shape; however, ED was sensitive to the change in the annulus size. The ED should be measured during the systole to capture the maximum annulus size.

Aortic valve calcification did not influence deformation during the cardiac cycle. Calcification of the cusps and aortic annulus was present in almost all patients and could be a further cause of paravalvular leaks, as eccentric calcification can prevent optimal deployment and adaptation of the prosthesis [20, 21].

A short distance between the aortic annulus and the coronary ostia (ostial height) can lead to occlusion of the coronary arteries [22, 23]. In our study population, the mean distances appeared to be safe for the implantation of the most common prosthesis types, which correlated with a clinically low risk of coronary obstruction. However, the variances showed that coronary ostia distances can be less than 5 mm for both, the LCA and RCA, indicating a potential risk of coronary obstruction [22]. Therefore, an exact measurement of the distances between the coronary ostia and the annulus appears to be essential, along with taking into account the LSC as an indicator of the sinus shape. Because no coronary obstruction occurred in our patients, we assumed that the LSC may have a positive effect on avoiding coronary obstruction. This conjecture should be clarified in further studies.

It is also important to note that the mean coronary distances are shorter in the systolic phase. This finding corresponds to the aforementioned effects of aortic root deformation during the cardiac cycle. In particular, there is a significant cycle-dependent difference in the distance between the annulus and the RCA.

In conclusion, the anatomy of the aortic root showed a high inter-individual variability and dependency on the cardiac cycle, which must be strongly considered during the patient’s evaluation and selection prior to TAVI to reduce complications. The systolic effective diameter (ED), provided by ECG-gated CT, represents an appropriate measure for pre-operative size selection of the TAVI prosthesis.

## Abbreviations

AASR: Aortic annulus sphericity ratio
AS: Aortic stenosis
ASE: Agatston score equivalent
AVA: Aortic valve area
CT: Computed tomography
ED: Effective diameter
LCA: Left coronary artery
LCC: Left coronary cusp
LSC: Lateral shift of the coronary ostia to the inner aortic annulus
NCC: Non-coronary cusp
RCA: Right coronary artery
RCC: Right coronary cusp
ROA: Regurgitant orifice area
TAVI: Transcatheter aortic valve implantation
TEE: Transesophageal echocardiography
